# Ecological Dynamics Impacting Bluetongue Virus Transmission in North America

**DOI:** 10.3389/fvets.2020.00186

**Published:** 2020-04-17

**Authors:** Christie Mayo, Emily McDermott, Jennifer Kopanke, Mark Stenglein, Justin Lee, Candace Mathiason, Molly Carpenter, Kirsten Reed, T. Alex Perkins

**Affiliations:** ^1^Department of Microbiology, Immunology, and Pathology, Colorado State University, Fort Collins, CO, United States; ^2^Entomology Branch, Walter Reed Army Institute of Research, Silver Spring, MD, United States; ^3^Office of the Campus Veterinarian, Washington State University, Spokane, WA, United States; ^4^Department of Biological Sciences, University of Notre Dame, Notre Dame, IN, United States

**Keywords:** bluetongue virus, evolution, ecology, epidemiology, infectious disease dynamics

## Abstract

Bluetongue virus (BTV) is an arbovirus transmitted to domestic and wild ruminants by certain species of *Culicoides* midges. The disease resulting from infection with BTV is economically important and can influence international trade and movement of livestock, the economics of livestock production, and animal welfare. Recent changes in the epidemiology of *Culicoides*-transmitted viruses, notably the emergence of exotic BTV genotypes in Europe, have demonstrated the devastating economic consequences of BTV epizootics and the complex nature of transmission across host-vector landscapes. Incursions of novel BTV serotypes into historically enzootic countries or regions, including the southeastern United States (US), Israel, Australia, and South America, have also occurred, suggesting diverse pathways for the transmission of these viruses. The abundance of BTV strains and multiple reassortant viruses circulating in Europe and the US in recent years demonstrates considerable genetic diversity of BTV strains and implies a history of reassortment events within the respective regions. While a great deal of emphasis is rightly placed on understanding the epidemiology and emergence of BTV beyond its natural ecosystem, the ecological contexts in which BTV maintains an enzootic cycle may also be of great significance. This review focuses on describing our current knowledge of ecological factors driving BTV transmission in North America. Information presented in this review can help inform future studies that may elucidate factors that are relevant to longstanding and emerging challenges associated with prevention of this disease.

## Introduction

Arthropod-borne viruses (arboviruses) constitute a significant group of emerging pathogens, many of which are increasing in global distribution as a result of climate change, urbanization, and changing of travel or trade (1–3). Bluetongue virus (BTV) is the etiologic agent of bluetongue (BT), an economically important arboviral disease of wild and domestic ruminants that is transmitted by various species of *Culicoides* midges (4–7). The expansion of *Culicoides-*transmitted arboviral diseases (BT, epizootic hemorrhagic disease [EHDV], Schmallenberg) on essentially every continent confirms that these diseases constitute a growing threat to ruminant communities (8–13). They also have substantial economic consequences. During an outbreak of EHDV in Israel in 2006, losses caused by reduced milk production and increased mortality were estimated at ~$2.5 million US dollars (USD), whereas the annual losses to the US livestock industry due to enzootic BTV infection were estimated at $144 million USD ~15 years ago (14, 15). In 2006, an unprecedented, multi-year epizootic of highly virulent BTV-8 began in Western Europe, outside of the assumed range of the disease. Country-specific costs associated with lost production, trade restrictions, control, and vaccination are estimated to be as high as 207€ million (16), and the cost per country to vaccinate animals alone is estimated in the tens of millions of Euros (17). Still, other *Culicoides*-borne threats loom that could be even more economically devastating, such as African horse sickness, which as the potential to severely impact the $122 billion USD US horse industry (18).

Novel BTV serotypes have also recently been identified in historically enzootic countries or regions including the southeastern United States (US), South America, Israel, and Australia, reflecting diverse means for spread of these viruses between regions (13, 19–22). Despite the link of climate change to recent incursions of BTV in Europe and expansion of geographic range in North America, there is limited information on how factors related to the ecology of BTV's vertebrate and invertebrate hosts might impact the evolution, distribution, and transmission dynamics of BTV (1, 23, 24). This is potentially important because a changing climate interacts with habitat and landscape variability to jointly determine opportunities for host-vector contact and the competence of vectors engaged in contact (25, 26). While transmission patterns of midge-borne viruses have been linked to heterogeneity in climate and land use worldwide, the role of host density and diversity in regulating the spread or viral evolution of these multi-host pathogens has been underappreciated (27–29). Identification and characterization of these ecological drivers could play a role in analyses to estimate the risk of BTV transmission and to inform appropriate strategies for control and prevention. In this article, we briefly summarize the current understanding of ecological factors driving BTV transmission within North America.

## Bluetongue Virus

BTV is within the genus *Orbivirus*, family *Reoviridae* (30). The BTV genome consists of 10 segments of double-stranded RNA (dsRNA), and each gene segment encodes at least one protein (31, 32). The BTV virion includes seven proteins (VP 1–7), and at least five additional non-structural proteins (NS 1,2,3/3A,4) that are produced in virus-infected cells (32, 33). The structural protein VP7 expresses group antigens common to all BTV strains and serotypes, whereas segregation of BTV into serotypes is largely determined by VP2 outer capsid protein (34–37). At least 29 serotypes are recognized worldwide, but the virus strains of the same serotype may have markedly different virulence even to highly susceptible ruminants (38–42). Genetic diversity is generated among field strains of the virus by both genome segment reassortment and mutation (43). Intrasegment recombination also can occur between virus strains, either within the vertebrate (ruminant) or invertebrate host (*Culicoides* midge) (43, 44). North American BTV isolates have been previously characterized by genotype based on segment 10 sequences (820bp region of the NS3 protein) (45). Although these analyses have provided key information into the relationships of BTV strains that circulate within the US and adjacent (such as the Caribbean Basin and Central America) and distant (such as Europe, Africa, Asia, and Australia) regions, there is a lack of comprehensive sequence data for all genomic segments. As a result, estimates of gene flow among field strains of BTV tend to be highly speculative. Similarly, the genetic determinants of viral phenotype that may impact spread and persistence, such as virulence, remain poorly characterized. Genome sequencing of field and laboratory strains of BTV has shown a high degree of segment reassortment resulting in the variety of currently circulating viral strains in the field, as compared to historic isolates, which could lead to amplification of viral transmission (46–49).

Emergence of a virulent virus (by reassortment or mutation) could stem from enzootic viruses that currently circulate in the US, or the translocation of a novel virus from an adjacent (Caribbean Basin, Latin South America) or distant (Asia, Europe, and Africa) region. In North America, BTV-2 was recently (2010) isolated in California, representing trans-continental dissemination of this virus serotype first described in the US in Florida in 1982 and that had previously been considered restricted to the southeastern US (21). The strain of BTV-2 isolated in California is a reassortant of BTV-2 and BTV-6, the latter a previously exotic serotype to North America (50). Similarly, strains of BTV-3 that have recently expanded their range beyond the southeastern US are able to readily reassort with BTV strains historically enzootic in the US (51). Recent studies based on BTV field isolates have shown reassortment is common and may drive phenotypic change resulting in a fitness advantage for the virus (46, 48, 49, 52).

Additionally, there is the issue of live attenuated vaccines being able to reassort with enzootic viruses contributing to the genetic backbone and potentially introducing novel biological properties of circulating viruses (53). Studies in both North America and Europe suggest that live-attenuated BTV vaccine viruses (or individual genome segments thereof) used to vaccinate livestock can be acquired and transmitted in the field by vector midges, thereby contributing to the gene pool of circulating viruses (54–57). Midge movement between vaccinated livestock populations and susceptible wild ruminant populations could drive viral evolution and reduce the efficacy of vaccination. Most of the major BTV vector species, including *C. sonorensis*, the primary US vector, feed opportunistically on large mammals (58), and are potential bridge vectors between livestock and wildlife populations.

Since the recent European BT epizootic, considerable focus has been placed on quantitatively defining aspects of Culicoides vector ecology and the genetic diversity of BTV strains (27, 59–61). Studies on the small-scale movement of *Culicoides* between farms and adjacent wildlife habitats, as well as on the frequency of contact between livestock and wildlife (e.g., deer and sheep sharing pasture) are needed to better understand BTV ecology. With the advent of next-generation sequencing and other technologies, quantifying within-host pathogen evolution is happening increasingly (62, 63). Acquisition of such information is pivotal for the future prediction of emergence and impact of *Culicoides*-disseminated viruses in divergent ecosystem contexts with transmission models.

## Distribution

The global distribution of BTV infection corresponds with that of competent *Culicoides* vectors and suitable environmental ecosystems and the range historically has been between 40–50°N and 35–40°S (5, 39). The global distribution of BTV has altered recently, perhaps as a consequence of the impact of climate change on *Culicoides* midges that serve as the biological vectors of the virus (1, 6, 23). In particular, since 1998 multiple BTV serotypes spread throughout the Mediterranean Basin and, in 2006, additional virus serotypes invaded and spread throughout extensive portions of northern Europe to precipitate an economically devastating epizootic (64–66). This epizootic was ultimately controlled in 2010 with an extensive vaccination campaign and use of inactivated vaccines; however, the re-emergence of BTV-8 in France in 2015 has caused speculation with regards to source or mechanism of viral introduction (67). Additionally, novel serotypes of BTV have recently invaded historically-enzootic countries (Israel, South America, and Australia) and non-enzootic countries (China, Republic of South Korea) (1, 11–13, 19, 22, 68). The expansion of novel BTV serotypes into these regions demonstrates the wide distribution of competent *Culicoides* species and, with the impact of climate change, it can be anticipated that new BTV strains and serotypes will continue to be introduced on a regular basis (40, 69).

Coincident with this invasion of novel serotypes in Europe and elsewhere, 11 previously exotic serotypes (serotypes 1,3,5,6,9,12,14,18,19,22,24) have been isolated in the southeastern US since 1999 (39). Four serotypes (serotypes 10,11,13,17) have long been enzootic throughout much of the US (40, 70, 71). While BTV serotype 2 has been considered enzootic in the US since its identification in 1982, infection was thought to be confined to the southeastern US (Florida and adjacent states) until the isolation of this virus in California in 2010 (21, 72). Most recently, BTV serotype 3 has spread throughout much of the US exhibiting extensive reassortment with genes of traditionally enzootic serotypes (BTV-10, 11, 13, 17) (51). Historically, national surveys conducted by United States Department of Agriculture (USDA)/Animal and Plant Health Inspection Service (APHIS) have utilized a threshold of <2% seroprevalence to differentiate between BTV-free and BTV-enzootic areas (73). The latest survey conducted from 1991 to 2004 concluded that BTV was enzootic in all states excluding Alaska, Hawaii, Michigan, Minnesota, New York, and Wisconsin (74). There is regional variation in the prevalence of BTV infection of livestock throughout the US (75–78). Additional serologic surveys of wild ruminant species have confirmed that several species (white-tailed deer, black-tailed deer, mule deer, elk, pronghorn, and bighorn sheep) are infected in enzootic areas (79). Although the strains of BTV that currently circulate in the US typically cause mild disease, epizootics of severe BT occur regularly amongst sheep and wildlife (white-tailed deer in particular).

Within North America, *Culicoides* midges were initially confirmed to be vectors of BTV by experimental infection of sheep using *Culicoides* midges collected during an outbreak of BT in Texas (80). At least two distinct and apparently stable BTV ecosystems have been identified in the Americas: one that includes Central and South America, the Caribbean Basin, and portions of the southeastern US, and a second area consisting of the remaining section of North America (72, 81, 82). *Culicoides sonorensis* is the predominant, if not exclusive, vector of BTV serotypes 10, 11, 13, and 17 across most of the US, south and west of the so-called “Sonorensis Line” which extends from approximately Washington State to Maryland (81). In the southeast US, *C. sonorensis* is rarely collected in areas with active BTV transmission (83), and so is not considered to be the primary vector in this area and the Caribbean. Although not conclusively proven to transmit BTV, several wildlife-associated species, including *C. stellifer* and *C. insignis*, are implicated in transmission in the southeast, as they are known to feed on livestock and wild ruminants and frequently test positive for BTV and/or EHDV (58, 83, 84). Many other *Culicoides* species known to feed on large ruminants are present within the US, but their contribution to the transmission of BTV remains uncertain (85). The absence of BTV in the northeast US appears to be due to a lack of a competent vector species. *Culicoides variipennis*, a sister species to *C. sonorensis*, is present in the northeast in livestock habitats, but either its vector competence or vectorial capacity are low enough that it apparently cannot support BTV transmission (81). Historical serological studies of ruminants in northern North America over many years have confirmed that climatic conditions prevent substantial virus transmission to ruminants (86).

*C. sonorensis* has also been recorded in parts of Canada, and BTV has periodically and transiently incurred in the Okanagan Valley, British Columbia, though these outbreaks appeared to be seasonal introductions without evidence of overwintering (87, 88). More recently, *C. sonorensis* was identified in Ontario on multiple farms during consecutive years (2013–14), suggesting established, overwintering populations (79). The discovery of *C. sonorensis* in Ontario was quickly followed in 2015 by the first recorded case of BTV in Canada outside of the Okanagan Valley, in an animal near where midges were collected (88). Although Canada was previously thought to be unable to support persistent *C. sonorensis* populations, the discovery of both virus and vector in Ontario suggests that a changing climate may be allowing a northward expansion of the disease.

## Transmission

### Inter-seasonality and Maintenance of Virus in Seasonally Enzootic Regions

The primary route of BTV transmission to its vertebrate (ruminant) host is through the bites of virus-infected hematophagous *Culicoides* midges which serve as biological vectors of the virus (5, 39, 58, 65). Although BTV is transmitted between ruminants in tropical regions throughout the year, infection is distinctly seasonal in temperate areas where the vast majority of infections occur during the late summer and autumn months (3, 76, 89–91). The virus largely disappears from resident ruminants in much of the Northern Hemisphere between early winter until mid-summer (mid-November until at least late July) (39). The precise mechanism of this highly seasonal nature of annual BTV infection remains poorly defined, including the relative contributions of animals and insects to the process of “over-wintering” (92). Even in subtropical regions of the US, *C. sonorensis* population density is seasonal, with peaks in mid to late summer and low abundance during the winter (93, 94). In these areas, *Culicoides* populations may persist trans-seasonally as long-lived adults with potentially some continued reproduction. Recent studies in California have confirmed the presence in mid-winter of BTV-infected parous female *Culicoides* midges without concurrent infection of adjacent sentinel cattle, suggesting that vectors infected in the prior seasonal period of transmission might sustain BTV throughout the over-wintering period in seasonally enzootic areas (93). Adult midges could survive for long periods during the winter months in farm buildings, or other habitats such as tree holes and vegetation, sporadically re-surfacing to feed on hosts or nectars from plant sources (93, 95). In temperate enzootic zones, like Colorado, freezing winter temperatures preclude adult activity, and it is thought that in these areas, *C. sonorensis* populations persist as overwintering larvae (81). However, laboratory experiments have shown that eggs are the most cold-tolerant life stage (96), and are also highly desiccation tolerant (97), suggesting that they may be the true overwintering stage. Although eggs may be how the vector persists, they are unlikely to be how the virus persists. Despite a single report of BTV RNA being detected in field-collected *C. sonorensis* larvae in the north-central US, subsequent studies have been unable to recover either RNA or live virus from *Culicoides* larvae, and transovarial transmission of virus has not been described in *Culicoides* spp. (98, 99).

Although the bite of infected *Culicoides* remains the primary source of BTV infection, transmission of BTV can occur independent of the vector. Some of the novel, small ruminant BTV strains (BTV-25, BTV-26, BTV-27) may be transmitted by contact (horizontally) without the involvement of *Culicoides* midges (100–102). Oral BTV infection of both ruminant livestock and wild and zoo carnivores has been described, including infection of calves by infectious colostrum (103–106). Vertical transmission of BTV in animals has been described, in particular with live-attenuated BTV and European BTV-8 vaccine strains (107–109). Lastly, the movement of BTV-infected animals may be responsible for translocation of BTV; however, these events are only relevant if the local vector population is competent and capable of transmitting the virus.

## Ecological Drivers of BTV Transmission

The dynamics of BTV transmission in multi-host ruminant systems are complex (110, 111), particularly with the additional complication of one or more vector species with heterogeneous host feeding preferences and contact rates (112, 113). Vertebrate host communities are variable in space and dynamic in time, making it particularly difficult to generalize about the impacts of host community structure on pathogen transmission. There may also be potential to confuse effects of host density and diversity on transmission given inherent challenges associated with experiments that have been performed to address effects of host diversity on pathogen transmission (110, 111). This ecological context is especially rich with arboviruses, which are subject to selective pressures in multiple host species and experience an environmentally sensitive stage in ectothermic vectors.

### Vertebrate Host

*Culicoides* feeding can cause physiologic and immunologic responses in mouse models resulting in the recruitment of leukocytic cells to bite sites (114). Recruitment of susceptible cell populations to the position of deposited virus occurs within hours of feeding and may explain a single infected midge's ability to transmit BTV to naive sheep with an efficiency of 80–100% (114, 115). BTV preferentially infects endothelial cells that line the walls of blood vessels, mononuclear phagocytic cells, and dendritic cells (114). After replication, BTV is released into the bloodstream where it interacts with blood cells (platelets, erythrocytes). Due to the intimate association of BTV with ruminant erythrocytes, viremia can be prolonged and is critical for transmission of the virus to susceptible *Culicoides* vectors via contaminated blood meals (116–118).

Virus-mediated damage to endothelial cells leads to vascular thrombosis, infarction of the tissue, necrosis, and hemorrhage (117, 119). The lesions of bluetongue are characterized by coronitis and laminitis, mucosal erosions, myonecrosis, subcutaneous and fascial edema, gastrointestinal ulceration, pulmonary edema, pericardial effusion, hemorrhage, ecchymoses and petechiae, and coagulopathy, among other features (38, 116). All ruminants are susceptible to BTV infection, but the most severely affected are sheep of European breeds (38). Bluetongue may also occur in other domestic and wild ruminant species (e.g., bighorn sheep, white-tailed deer, pronghorn, etc.), but severe clinical BT was rarely described in cattle prior to the 2008 BTV-8 epizootic in Europe (120, 121). Within enzootic regions such as the US, disease tends to be subclinical although sporadic epizootics have occurred. The most significant epizootic reported within the last two decades occurred throughout southern Montana and Wyoming during November of 2007 (122). Over three-hundred domestic sheep died as the result of BTV-17 infection that also affected wildlife populations of pronghorn antelope, white-tailed deer, and mule deer (122).

The ability for BTV to result in an epizootic requires the virus to overcome significant barriers. Aside from a susceptible host's physical presence, BTV needs to evade the ruminant host's adaptive and innate immune responses. Infected animals respond with interferon production to BTV infection, and both humoral and cell-mediated immune responses (123). The VP2 outer capsid protein induces serotype-specific neutralizing antibodies and provides protection against reinfection with homologous virus serotypes with minimal cross reactivity between serotypes (124–126). Such antibodies are detected about 2 weeks after natural infection and can last for up to 4–6 years (106, 127). Once an animal has developed immunologic memory to BTV serotypes circulating within a region, herd immunity can provide a limitation for viral infection and should be considered during epidemiologic investigations and surveillance strategies.

BTV's intimate association with erythrocytes facilitates both sustained ruminant infection and infection of *Culicoides* vectors that feed on viremic ruminants (128). With regards to disease ecology, this unique feature inevitably determines its potential for enzootic stability and geographic spread. The duration of viremia is highly variable among different ruminant species. Although the duration of viremia in cattle ranges from 7 to 63 days based on virus isolation, the maximum duration in other wild ruminants has varied by species: 17 days in blesbok (*Damaliscus dorcas*); 41 days in bison (*Bison bison*); 22–28 days in white-tailed deer (*Odocoileus virginianus*);10 days in North American elk (*Cervus elaphus*), and 35 days in mountain ganzelle (*Gazella gazella*) (118, 129–131). The variability in duration of viremia reported in these species can depend on the virus serotype, blood fraction examined, and virus detection system used. Ultimately, the duration of viremia in BTV-infected ruminants that is infectious to vector insects is a prerequisite to understanding disease transmission and ecology.

### Invertebrate Host

After BTV-infected ruminant blood is ingested by a competent female *Culicoides* midge, it passes with the blood meal into the lumen of the hind portion of the midgut. From there, the virus must pass several epithelial tissue infection and escape barriers to infect, disseminate within, and be transmitted by the insect. In the midgut, viral infection and multiplication occurs in the mesenteronal cells, followed by release of progeny virus into the hemocoel (dissemination). Recent studies have identified a functional response to RNAi in KC cells derived from *C. sonorensis* which are successful in inhibiting BTV infection (132). While other studies have demonstrated that putative RNAi pathway members exist in *C. sonorensis*, it is unclear how these interactions limit viral replication within the invertebrate host (133). Successful release of BTV into the hemocoel allows for transit and subsequent multiplication in multiple tissues and organs. In a disseminated infection, BTV infects nearly every tissue in the insect's body, with the exception of the reproductive tissues, and this likely explains the lack of observed vertical transmission in *Culicoides* (99). Infection of the salivary glands, and escape into the salivary gland lumen, is required before the vector can transmit BTV to a susceptible vertebrate host during blood feeding. The time required to achieve this cycle is called the extrinsic incubation period (EIP), and lasts, on average, between 1 and 2 weeks, but the range can vary substantially depending on environmental conditions (primarily temperature) (99).

## Environmental Influences

As with all vector-borne diseases, the natural transmission cycle of BTV is dependent on relationships between the pathogen (BTV), vector (*Culicoides* spp.), and host (ruminant). Many of these interactions can be influenced by environmental and anthropogenic factors (134). A mathematical quantity known as vectorial capacity has been established to better estimate the relative capability of a vector population to transmit a pathogen to a population of susceptible hosts (135–137). This quantity is defined as:

$$\begin{array}{l} C=\frac{ma^{2}Vp^{n}}{-lnp}, \end{array}$$

where *C* = vectorial capacity, *m* = vector-host ratio, *a* = bites per vector per day, *V* = vector competence (suitability of the vector population for pathogen infection and transmission), *p* = the daily probability of survival of the vector, and *n* = the extrinsic incubation of the pathogen (136). An important limitation of this formulation is that, in reality, its components are altered by fluctuating environmental factors. Due to the complexity of the factors that influence vectorial capacity, few studies have attempted to calculate it based on field data for any pathogen. Gerry et al. (136) estimated the vectorial capacity of *C. sonorensis* on a southern California dairy farm by experimentally measuring all components of the equation in the field. Their model was predictive of sentinel calf seroconversions in 2 of 3 years. Different components of the vectorial capacity equation may be more or less influential in the outcome in different vector-pathogen systems. For BTV, host biting rate may have the greatest effect on vectorial capacity and is heavily influenced by temperature (136).

While a variety of factors (humidity, food quality, and adult body size) can influence seasonal activity of *C. sonorensis*, temperature remains one of the most predictable variables in determining fluctuations of the total population (87, 136, 138). Within temperate regions such as North America, the greatest abundance of adult *C. sonorensis* populations occurs with temperatures ranging from 28 to 30°C (77, 94, 139, 140). Temperature affects the daily survival probability, *p*, the biting rate, *a*, and the ratio of vectors to hosts, *m*, through shorter generation times. A four-degree increase in temperature (13–17°C) was associated with a 5 day decrease in egg development time. Fecundity in females held at 13°C was also significantly lower than in females held at temperatures of 17°C or higher (141). The increase in reproductive output associated with higher temperatures is somewhat offset by a decrease in daily survival at temperatures above 20°C (142, 143). However, it is important to note that the environmental air temperature measured in the field may not be equivalent to the actual temperature experienced by midges. *Culicoides* are crepuscular insects and likely rest in shaded or otherwise protected locations during the heat of the day, and the temperature in these microhabitats may be much lower than the overall air temperature. The number of adults generated per season is also dependent on the development and survival of immature midges, which are sensitive and directly related to temperature (144).

### Vector Survivorship and Larval Development

As with adult midges, warmer temperatures reduce the time of development for the four larval instar stages allowing pupae to emerge at a faster rate. Increasing the temperature from 20 to 30°C reduced *C. variipenniis* larval development time from first instar to pupation by 9.0 days for a New York population (141) and 17.8 days for a Virginia population (145), but the speed of emergence during warmer conditions compromises the size and fecundity of newly emergent female *Culicoides* (141, 145). Small body size is also associated with a higher susceptibility to viral infection in mosquitoes (146).

Recent studies have identified that habitat suitability for *C. sonorensis* is associated with a number of biotic factors including temperature, land use, distribution of hosts, and Normalized Vegetation Index (23, 27, 29, 147). However, the relationship of these variables to *C. sonorensis* populations remains poorly defined in North America. Additional environmental factors that support larval populations of *C. sonorensis* include standing or slow-moving, sunlight-exposed aquatic environments, especially those contaminated with manure (148, 149). *C. sonorensis* larval habitats commonly have higher salinity concentrations than those of *C. variipennis* (148). Dramatic fluctuations in precipitation can indirectly affect the development of immature *C. sonorensis* by providing alterations in breeding habitat, but temperature remains the most influential factor affecting their development rates (150, 151). While *C. sonorensis* is typically associated with man-made livestock habitats, the putative BTV vectors in the southeast US (e.g., *C. stellifer* and *C. insignis*) are often found in sylvatic environments. Studies on the ecology of other BTV vectors in North America are far less numerous than those on *C. sonorensis*, but recent work indicates that standing water and stream margins support the development of species such as *C. hematopotus, C. stellifer*, and *C. venustus* (152).

### Vector Competence to BTV

Vector competence of adult midges is genetically determined but environmentally influenced. Susceptibility to BTV infection is specific and may vary between *Culicoides* species, serotype of BTV ingested, or even geographical populations of *Culicoides* of the same species (153). The infection and dissemination process can be complicated by genetic (midgut infection and escape barriers) or temperature influences (148, 154). For example, optimal BTV transmission by *C. sonorensis* occurs at high temperatures 27–30°C, whereas the virus is unable to develop at temperatures below 14–15°C. As temperatures increase, virogenesis increases in a competent vector (153, 155). Due to increased virogenesis at higher temperatures, the extrinsic incubation period varies dramatically based on ambient temperature fluctuations. Studies have identified that the duration to reach peak virus titre is 4–8 days when *C. sonorensis* are held at 27–30°C, whereas it can take 16–22 days for titer to peak when held at 21°C (91, 153). Therefore, transmission of BTV is optimized during periods of warmer weather, at least above a threshold level of ~9–15°C, when the extrinsic incubation period has shortened sufficiently to permit transmission within the lifespan of *C. sonorensis* (91, 138, 153).

Temperature may have additional effects on *Culicoides* competence for BTV beyond increasing the speed of viral replication. *C. nubeculosis* is considered refractory to BTV infection under normal conditions; however, when larvae are held at high temperatures during their development, a proportion (~10%) of those individuals can become infected with African horse sickness virus as adults (156). It has been suggested that high temperatures may damage the midgut epithelium such that viruses are able to bypass the midgut infection barrier and pass directly from the gut lumen into the hemocoel, thereby allowing non-vector species to transmit arboviruses, and that climate change may increase the frequency of these events (157).

## Anthropogenic Influences

Beyond temperature, the ecology of BTV transmission is highly unpredictable, as many other factors can serve as drivers of transmission. Studies have quantified the effect of measurable parameters such as temperature, wind speed, or precipitation on BTV transmission in invertebrate and vertebrate hosts, but most of these studies are limited to work with a single vector species, or used *Culicoides* reared in laboratory conditions (64, 147, 158–160). While important, these studies cannot account for the ecological interactions occurring in the field due to anthropogenic confounders, like land use and animal husbandry practices. These anthropogenic influences are difficult to measure, both locally and globally, and it is challenging to assess the tangible effect humans have had or will have on the transmission cycle of BTV. On the largest scale, there is a great deal of uncertainty as to how climate volatility will be either associated or driven by anthropogenic influences altering the dispersion of arboviral diseases (147, 161).

Many authors have suggested that pathogen-vector-host relationships may be affected by landscape alterations that contribute to changes in the conditions of vector breeding. Although *C. sonorensis* is stereotypically considered to develop primarily in livestock wastewater ponds, these midges would have evolved to develop in more transient water sources associated with wild ruminant species. In the plains states, *C. sonorensis* larvae can be found in active bison wallows, which share features with artificial wastewater ponds that make them appropriate for development: gentle slopes, free from vegetation, and enriched with animal manure (162). These wallows are temporary puddles, and the transient nature of this resource would naturally limit midge population sizes. Wastewater ponds, on the other hand, are largely permanent water sources, providing excellent year-round development sites for *C. sonorensis*, and encouraging high levels of vector-host contact (140). On individual California dairy farms, these local management/land use practices are necessary to operate a successful and profitable business. However, these practices also increase the processes that result in the intersection of multiple natural and anthropogenic landscapes. Ecotones were originally identified as specialized wildlife habitat characterized by readily identifiable edges or transitions zones between major types of vegetation (134, 163). Recent definitions have described ecotones as a dynamic process where constituents of ecological systems influence biodiversity and ecosystem function (164). This expands the definition of ecotones as areas where biophysical factors, biological activity, and ecological evolution are associated and may be intensified (164). Recent studies have suggested that the occurrence of BT and associated *Culicoides* populations could be related to either landscape features (forest, open pasture, and areas without vegetation) or host distribution. However, little information is provided as to a clear mechanism by which landscape indices could influence BTV circulation (23, 147, 165, 166).

By concentrating large populations of vectors near large populations of suitable hosts, BTV transmission is likely intensified in livestock systems compared to normal sylvatic cycles. This intensification provides suitable opportunities for BTV to infect multiple vertebrate and invertebrate host species, and genetically reassort genome segments which may lead to increased virulence. It can be concluded then that human activity has resulted in alterations of the spatial hierarchy of BTV infection leading to “ecotones within ecotones” from local habitat (enzootic California dairies) to the global biome (Northern Europe epizootic). Two principal points remain to be thoroughly characterized with regards to the influence of anthropogenic factors on BTV transmission: local management and landscape scale alterations (i.e., forest edge habitat, wastewater lagoons), and larger scale climate alterations from anthropogenic influences [i.e., climate change secondary to greenhouse gas emission; (167–170)].

## Epidemiologic Surveillance Strategies Informing Predictive Models

Better characterization of the environmental and anthropogenic drivers of emergence of BTV infections is clearly a prerequisite to predicting future occurrence and distribution of the disease, and to its control (10, 142, 171, 172). Surveillance in the veterinary community is described as surveying the occurrence of a disease and its status in the animal population. Entomological surveillance for vector-borne diseases involves collecting insects from the environment using mainly passive traps, and testing the species most likely to be infected with the pathogen of interest in pools of multiple individuals. Most often this is done to obtain basic information about a disease (incidence, prevalence, transmission, enzootic presence, and epizootic spread) required to inform statistical analyses and ultimately guide policy makers to make informed decisions for mitigation strategies or policy change. Therefore, it is critical to understand the questions driving the purpose of surveillance before designing strategies to achieve those goals (159, 173, 174). Many countries focus their surveillance efforts on diseases notifiable to the World Organization for Animal Health (the OIE); however, the collection of surveillance data, regardless of criteria for reporting, can be an invaluable first step and critical when developing accurate models to predict risk and assess mitigation strategies for the future (174, 175).

### Vertebrate Host

Surveillance systems utilized to monitor BTV among ruminant hosts in North America have largely relied on disease reporting or periodic cross sectional testing of sera from slaughter cattle or the monitoring of hunter-killed white-tailed deer (74, 75, 122). While additional surveillance has been conducted within individual states of the US, there are limited nationwide strategies to account for both the temporal and spatial variation of BTV infection among ruminants and vectors within a given season (77, 78, 160, 176, 177). Sentinel animal surveillance, as recommended by the OIE code, provides a useful tool in monitoring arthropod populations and viral infection rates of sentinel ruminant hosts in order to detect arboviral activity. The advantage to this targeted surveillance vs. other forms of random surveillance is the ability to act as an early warning system. Many countries experiencing both enzootic (e.g., Australia) and epizootic (e.g., Switzerland) cycles of infection utilize sentinel animal surveillance as a successful monitoring tool to advise their models for decision support and mitigation strategies [e.g., info-gap theory, scenario tree modeling; (173, 174, 178)].

### Invertebrate Host

As with vertebrae host surveillance, one of the most critical points in invertebrate surveillance is assessing the goals of the program. There are many ways to categorize an insect population but optimal trapping systems have been developed to characterize species of insects present in a particular geographic location in order to: (1). examine host associations of animal-biting insects; (2). assess the seasonal activity or geographic distribution of insect species, (3). or measure parameters of pathogen transmission including host feeding preference, pathogen infection prevalence, or host biting rate (bites per host per time) (93, 154, 179–182). Animal-baited trapping provides the most useful tool when researchers are seeking to understand arboviral transmission in a natural ecosystem (136, 181, 183, 184). Host biting rate is of particular interest in the transmission cycle, as one would expect that an increase in biting rate would result in increased pathogen transmission to susceptible hosts (136, 185). Accurate measurement of biting rate can be difficult for hematophagous insects even when using animal-baited aspiration methods, but these methods provide the most accurate information in the field setting. Some rather important limitations include enclosures or aspirations that may trap biting insects in addition to those simply attracted to the vicinity of the host but not feeding (186–188). Assessing engorgement rate and parity of these species can provide additional information with regards to feeding status, but other complications include competition of the human collector who stands in close proximity to the target host.

Besides the biological considerations, use of bait animals is labor- and cost-intensive, and there are inherent risks of injury to the handlers or animals themselves. Therefore, other trapping devices are most often used in field surveillance. These traps [e.g., New Jersey light trap, CDC miniature light trap, encephalitis vector/vector surveillance (EVS) trap] are either baited with artificial semiochemicals (e.g., CO_2_) or light of an appropriate wavelength (e.g., UV), and capture a diverse subset of the *Culicoides* population. However, these trap designs may reduce the overall capture of insects at individual locations compared to animal-baited aspiration, as observed in southern California, where *C. sonorensis* abundance was 3.7 times greater when captured from “bait” cattle than from suction traps baited with CO_2_ (136). Furthermore, subsequent studies demonstrated that BTV field infection rates in *C. sonorensis* were lower in insects collected by suction traps baited with both CO_2_ and UV light vs. traps baited with CO_2_ alone, suggesting that light actually repelled infected midges (184, 189). Further evaluation of viral dissemination within *C. sonorensis* demonstrated strong signal for viral deposition within the cornea and rhabdom (189). These structures within the compound eye are responsible for collecting and focusing light to form images; therefore, it was hypothesized that viral damage reduced *Culicoides* visual acuity, with subsequent changes in behavioral phenotypes (189). Pathogen manipulation of *C. sonorensis* behavior toward visual cues is supported by transcriptome analyses showing significant downregulation of genes known to be involved in sensory processes, particularly vision, after infection with EHDV, as well as downregulation of genes associated with memory and startle responses (190). It is unknown whether this effect is adaptive to BTV transmission, or whether it is a side effect of disseminated viral infection (189, 190). Therefore, the use of traps baited with UV light may provide a poor estimate of biting rate and lead to a misunderstanding of pathogen transmission, factors of critical importance when modeling risk of BTV transmission.

## Future Research Needs

Host-vector-virus transmission systems are dynamic and complex, with a variety of ecological drivers. Knowledge of the mechanisms driving the emergence or incursion of BTV from its natural maintenance cycle is still very limited. Studies have reported that maintenance and distribution of BTV is attributed to biotic factors, whereas patterns of vector behavior and abundance are likely related to abiotic factors (165). Understanding and defining these interactions is critical to predicting the occurrence of BTV infection of livestock through comprehensive determination of the impact of these drivers on vector abundance, competence, and vectorial capacity (87, 138, 191). Individual species of *Culicoides* midges require distinct and specific conditions for breeding, which could explain in part the increased rate of BTV transmission within certain geographic areas (180, 192). However, few studies to date have attempted to specifically address how biotic and abiotic drivers of infection are related to abundance of vectors and virus transmission among ruminants in North America.

Many questions about bluetongue remain, in part because of a lack of data and in part because of the overwhelming complexity of the studies that are necessary to capture important features of the ecology and evolution. Although data collection and accessibility are developing, more precise data are necessary to uncover some of the most pressing mysteries of this particular arbovirus. Identifying mechanisms for defining interactions among ecological drivers, host diversity, and the emerging risk of vector-borne diseases within ruminant communities could offer new insights into understanding the ecology of this virus. The domestic animal-wildlife-human interface is becoming an increasingly greater concern as a result of habitat fragmentation and land-use change. The ultimate goal is to provide tangible outcomes for predicting risk and mitigating vector-borne disease, particularly in the face of climate variability. These questions establish context for developing innovative ecological studies linking processes across multiple scales and have the potential to inform cost-effective, science-driven approaches to the development of mitigation strategies.

## Author Contributions

CMay, EM, JK, MS, JL, CMat, MC, KR, and TP made equal contributions to this review article.

## Conflict of Interest

The authors declare that the research was conducted in the absence of any commercial or financial relationships that could be construed as a potential conflict of interest.
